# The gut microbiome differs between hygiene-performing and non-hygiene-performing worker honey bees

**DOI:** 10.1007/s00040-025-01029-x

**Published:** 2025-03-06

**Authors:** Y. H. Tola, K. Wagoner, M. K. Strand, O. Rueppell, D. R. Tarpy

**Affiliations:** 1 Department of Applied Ecology, North Carolina State University, Raleigh, NC 27695-7617 USA; 2 Department of Biology, University of North Carolina Greensboro, P. O. Box 26170, Greensboro, NC 27402 USA; 3 Biology and Biotechnology Sciences Branch, U.S. Army Research Office, DEVCOM-ARL, Research Triangle Park, NC 27703 USA; 4 Department of Biological Sciences, University of Alberta, Edmonton, AB T6G2L3 Canada

**Keywords:** Apilactobacillus kunkeei, Bartonella apis, Frischella perrara, Gut microbiome, Hygienic behavior, Honey bee

## Abstract

Gut microbiomes play a significant role in the health, development, and behavior of numerous species, including honey bees (*Apis mellifera*). Worker honey bees exhibit varying degrees of hygienic behavior, which involves the removal of unhealthy brood to mitigate disease within their colony. However, the potential relationship between hygienic behavior and the honey bee gut microbiome has not been previously investigated. In this study, we compared gut microbiota in honey bees engaged in hygienic behavior (hygiene performers) versus those not exhibiting this behavior (non-hygiene performers) using 16S rRNA gene amplicon sequencing. Proteobacteria, Firmicutes, and Actinobacteria were identified as the predominant phyla. Notably, three bacterial species (*Apilactobacillus kunkeei*, *Bartonella apis,* and *Frischella perrara*) were found to be more abundant in hygiene performer bees compared to non-hygiene performer bees. Additionally, hygiene performer bees showed a higher diversity of amplicon sequence variants, with *Apibacter mensalis* being exclusively present in hygiene performer bees and absent in non-hygiene performer bees. These findings reveal an association between gut microbiota composition and hygienic behavior in honey bees, which may provide a foundation for future research exploring probiotic development and other strategies to enhance honey bee health.

**Supplementary Information:**

The online version contains supplementary material available at 10.1007/s00040-025-01029-x.

## Introduction

Honey bee (*Apis mellifera*) workers harbor a simple and conserved gut community of 8–10 bacterial phylotypes that make up 95–98% of their gut microbial community (Martinson et al. 2011; Moran et al. 2012). Five of these phylotypes are core members that are typically found in every adult worker bee (*Bifidobacterium, Bombilactobacillus* Firm-4, *Gilliamella, Lactobacillus* Firm-5, and *Snodgrassella*), while non-core phylotypes include *Apibacter*, *Bartonella, Bombella, Commensalibacter*, and *Frischella* (Kešnerová et al. 2016; Kwong et al. 2017). Within each of these phylotypes, more than one species has been characterized (Kwong and Moran 2013; Ellegaard and Engel 2019). Newly emerged bees acquire and establish gut bacteria by oral ingestion during trophallaxis and by fecal–oral transmission during social interactions with nestmates and through contact with hive substrates (Powell et al. 2014). Nevertheless, variation in microbiome composition among nestmates exists and the functional significance of this variation is not sufficiently understood.

The gut microbiome of honey bees plays a critical role in shaping various host phenotypes, including neurophysiological functions (Liberti et al. 2022; Zhang et al. 2022c, b; Cabirol et al. 2023), onset and intensity of foraging behavior (Vernier et al. 2024), weight gain (Zheng et al. 2017), immune responses (Emery et al. 2017), and social interactions (Vernier et al. 2020). These microbiome-mediated effects are modulated by hormonal signaling and are intricately linked to physiological and behavioral alterations, encompassing variations in diet, gene expression, body mass, and microbial community structure (Whitfield et al. 2003; Vance et al. 2009; Kather et al. 2011; Jones et al. 2018; Kešnerová et al. 2020). Understanding these interactions is crucial for elucidating the complex mechanisms underlying honey bee health and behavior.

Honey bee colonies face numerous disease agents that threaten their health and survival, including viruses, bacteria, fungi, and parasites (Evans and Schwarz 2011). Among the many behaviors that social insects employ to reduce disease at the colony level (Social immunity, reviewed by Cremer (2019)) is hygienic behavior, which involves the detection, uncapping, and removal of unhealthy (diseased or parasitized) brood from the hive (Boecking and Drescher 1992). Hygienic behavior reduces pathogen load at the colony level, thereby enhancing colony disease resistance and survival (Erez et al. 2022). However, given the physical contact and cannibalism involved, hygienic behavior likely exposes individual hygiene performers to a higher risk of pathogen exposure (Posada-Florez et al. 2021). While hygienic workers' increased exposure to pathogens in diseased brood may negatively affect their individual health and survival, their role in removing unhealthy brood from the colony may have a net positive impact on colony health and survival.

An altered gut microbiome in hygienic workers may reflect immune responses to heightened exposure to specific pathogens or could result from exposure to diseased brood. Evidence shows that gut microbiomes can stimulate host immune system function (Emery et al. 2017), enhance protection against specific pathogens (Daisley et al. 2020a; Steele and Moran 2021), and be altered by host exposure to pathogens (Rouzé et al. 2019; Ye et al. 2021; Lau et al. 2024). However, these studies have not specifically investigated the relationship between microbiota and hygienic behavior. Findings suggest that alterations in the microbiome may represent an adaptive response to environmental or behavioral changes, though the association between the gut microbiome and hygienic behavior remains unclear. Interestingly, previous research has linked gut microbiome composition to neurotransmitter levels in the honey bee brain (Liberti and Engel 2020; Zhang et al. 2022c; Cabirol et al. 2023), suggesting a possible connection between microbiome and host behavior. Similar mechanisms observed in other species support the hypothesis of an indirect link between gut microbiome and behavioral phenotypes (Liu et al. 2019; Nadeem et al. 2019).

While gut microbiomes have been shown to influence various honey bee behaviors, the relationship between gut bacterial communities and social immunity remains underexplored. In this study, we compared the gut microbiomes of honey bees that perform hygienic behavior (hygiene performers) with those that do not (non-hygiene performers). Our analysis revealed significant differences in microbial community composition between the two groups, with hygiene performers exhibiting a higher relative abundance of certain gut bacteria. These findings highlight associations between gut microbiome and hygienic behavior, providing a foundation for future research into the potential role of gut microbiomes in honey bee social immunity.

## Materials and methods

### Honey bee sample collection and preparation

Hygiene-performing and non-hygiene-performing worker honey bees were collected from two colonies determined to be hygienic via the UBeeO assay, previously described by Wagoner et al. (2021) and Wagoner (2023). Briefly, a small area of capped brood cells was treated with synthetic unhealthy brood odors (UBeeO) and the percent manipulation of treated caps was measured after 2 h. Colonies selected for this experiment were located at the primary UNCG apiary. Both experimental colonies scored above the 60% hygiene threshold required to be considered high UBeeO colonies (colonies A2H and A18H had UBeeO scores of 71% and 91%, respectively). Thus, despite the difference in UBeeO scores, the two experimental colonies have the same categorical hygiene classification, and thus similar hygienic performance expectations. To collect hygiene-performing and non-hygiene-performing worker bees, a second UBeeO assay was conducted in each colony. Experimental frames were selected based on the presence of numerous bees and large areas of capped and uncapped brood. Approximately 1 h into the assay, UBeeO-treated frames were collected from each colony. Ten nurse bees observed performing hygienic uncapping or removal behaviors (hygiene performers: HYGP) were collected directly from the UBeeO assay area of experimental frames. Another ten nurse bees located on the brood nest but that were not observed performing hygienic behavior (non-hygiene performers: NHYGP) were then collected from outside of the UBeeO assay area on the same frames. These NHYGP were confirmed to be nurses according to their task performance; only bees performing non-hygiene related nursing activities such as feeding larvae or capping fifth larval instars were collected as NHYGP (control) nurses. Rapid and accurate sample collection were facilitated by the specificity of the frame area from which hygiene-performing bees were collected and the large size of the brood nest and number of nurse bees present on the frame area from which the non-hygiene-performing bees were collected. Hygiene-performing and non-hygiene-performing bees (*n* = 40 total) were then individually washed in 4% sodium hypochlorite, followed by a wash in 70% ethanol and finally 1 × PBS for 2 min to eliminate any external microorganisms (Moran et al. 2012). The entire gut was dissected aseptically from each bee using forceps and gut placed in a 2 mL microcentrifuge-tube containing 500 µL PBS. Samples were stored at -80 °C until DNA extraction.

### DNA extraction and 16S rRNA amplicon sequencing

DNA was extracted using a CTAB Phenol chloroform method (Tola et al. 2020), where the entire gut was homogenized in a CTAB solution with β-mercaptoethanol and beads. The homogenate was incubated with phenol at 64 °C, followed by sequential chloroform and phenol–chloroform–isoamyl alcohol extractions. DNA was then precipitated with ethanol, washed, dried, and resuspended in MilliQ water. The 16S rRNA gene amplicon library preparation and sequencing were conducted at the UNC Microbiome Core Facility (School of Medicine at Chapel Hill, NC 27599–7032, USA, http://www.med.unc.edu/microbiome/, accessed on June 19, 2023).

### 16S rRNA gut community analysis

Raw amplicon sequences were analyzed using the DADA2 pipeline (Callahan et al. 2016) embedded in QIIME 2 (version 2019.10). Reads were checked for quality and primer sequences trimmed using “cut adapt” (version 2.10), and taxonomic classification was performed using the SILVA138 database using a pre-trained Naive Bayes classifier (Wang et al. 2007). In addition, local BLASTn searches against the NCBI 16S microbial database were performed. Amplicon sequence variants (ASVs) with a cumulative abundance below five across all samples, along with unwanted sequences of animal and fungal origin (e.g., Chloroplast, Chloroflexi, Mitochondria, Archaea, and Eukaryota), were discarded from the analysis. To minimize potential biases caused by variation in 16S rRNA gene copy numbers across bacterial taxa, we normalized relative abundances using the Ribosomal RNA Database (rrnDB, version 5.9; Stoddard et al. 2015). We conducted differential abundance analysis using the Analysis of Composition of Microbiomes with Bias Correction (ANCOM-BC) method to assess differences in microbial community composition between HYGP and NHYGP (Lin and Peddada, 2020, 2024). ANCOM-BC is particularly advantageous for microbiome data, which are often compositional, sparse, and contain structural zeros—challenges that traditional methods fail to address adequately. The method identifies differentially abundant taxa by analyzing log ratios of each taxon relative to others, thus accounting for the compositional nature of the data. ANCOM-BC minimizes spurious correlations through pairwise log-ratio comparisons and controls for multiple comparisons using False Discovery Rate (FDR) adjustments. This approach enhances the reliability and precision of detecting subtle differences in taxa abundance. Additionally, alpha diversity was assessed using Evenness, Faith’s Phylogenetic Diversity (Faith PD), observed features, and Shannon indices to evaluate microbial community richness and evenness. The data were rarefied to a minimum sequencing depth. Statistical comparisons were conducted using the Kruskal–Wallis test, followed by Dunn’s pairwise comparisons with Bonferroni correction. Boxplots were generated using the ggplot2 package (version 3.5.1) in R (version 4.3.2, Vienna, Austria) to visualize differences in bacterial species abundance and alpha diversity between hygiene performers and non-performers.

## Results

### Gut bacterial communities in hygiene performers vs non-hygiene performers bees

**Fig. 1:**
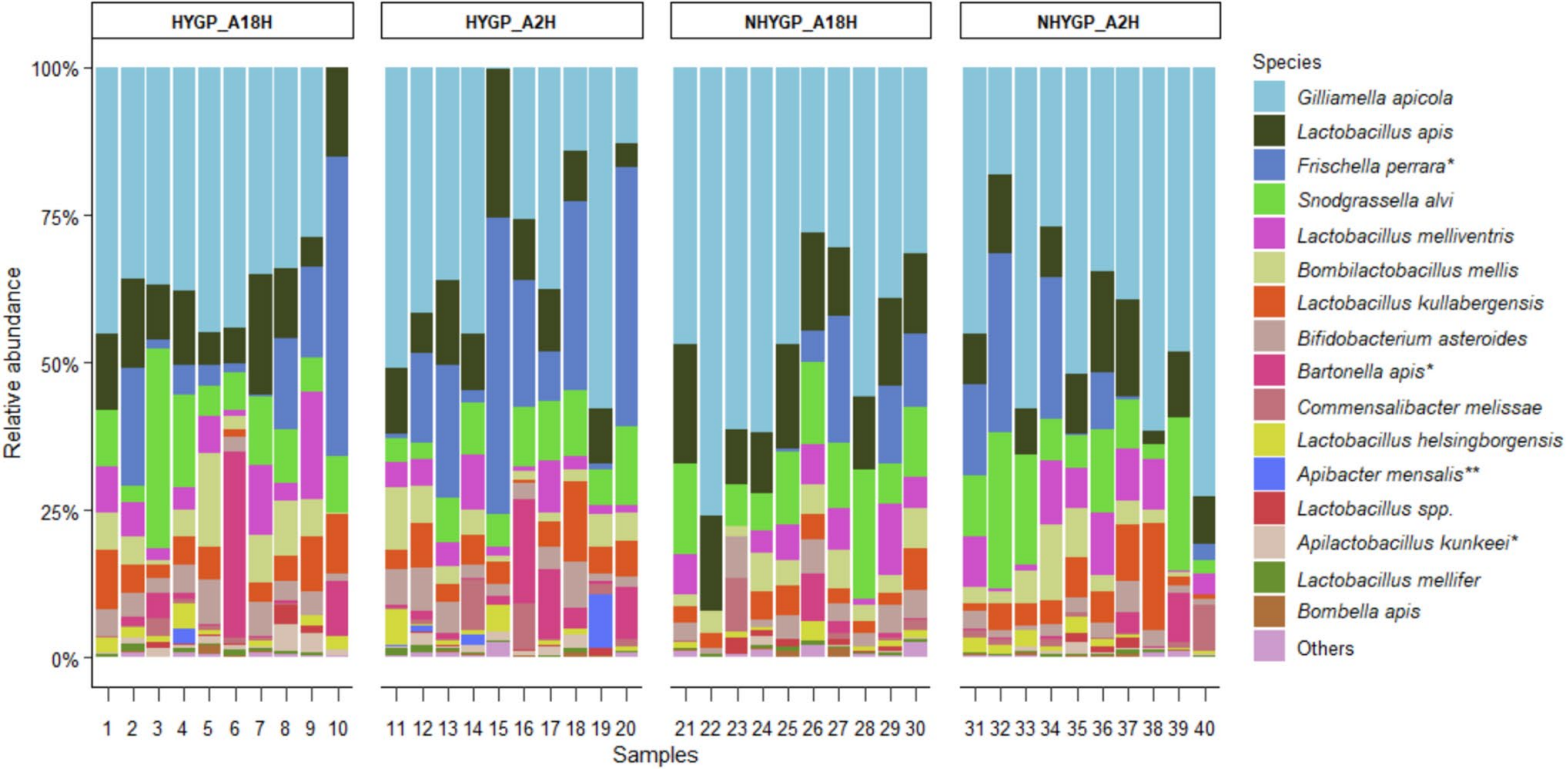
Gut bacterial species in hygiene-performing (HYGP) and non-hygiene-performing (NHYGP) worker honey bees. The relative abundance of bacterial species is shown for individual samples, categorized by colony (A18H and A2H) and hygiene group (HYGP and NHYGP). Bacterial species with significantly higher relative abundance in HYGP are denoted with an asterisk (*), while those found exclusively in HYGP are marked with a double asterisk (**). Species with an overall relative abundance of less than 0.2% are grouped together and labeled as "Others."

The 16S rRNA gene sequences were analyzed for gut samples to characterize bacteria associated with HYGP and NHYGP. We obtained 3,882,345 paired-end sequences of the V4 region of the 16S rRNA gene overall, with an average of 97,059 reads per sample (individual gut, number of reads ranged from 48,175 to 228,093). In total, we found 93 bacterial species, with 16 species representing more than 99.5% of total reads (Fig. 1, S1).

The gut bacterial taxa previously found to dominate the gut community of honey bees were represented in high proportions in our samples (Fig. 1). As expected, Proteobacteria, Firmicutes, and Actinobacteria were the dominant phyla identified in HYGP and NHYGP samples. The most abundant was Proteobacteria with about 69% of total reads encompassing *Gilliamella apicola, Frischella perrara*, *Snodgrassella alvi, Bartonella apis, Commensalibacter melissae,* and *Bombella apis. Gilliamella apicola* alone accounted for about 40% of total reads, followed by Firmicutes with about 28% of total reads including *Lactobacillus apis, L. melliventris, Bombilactobacillus mellis, L. kullabergensis, L. helsingborgensis, Apilactobacillus kunkeei,* and* L. mellifer,* among others. *L. apis* accounted for about 12% of total reads. Finally, Actinobacteria including *Bifidobacterium asteroides* represented about 3% of total reads.

### Differences in gut microbial community composition between hygiene performers and non-hygiene performers

**Fig. 2:**
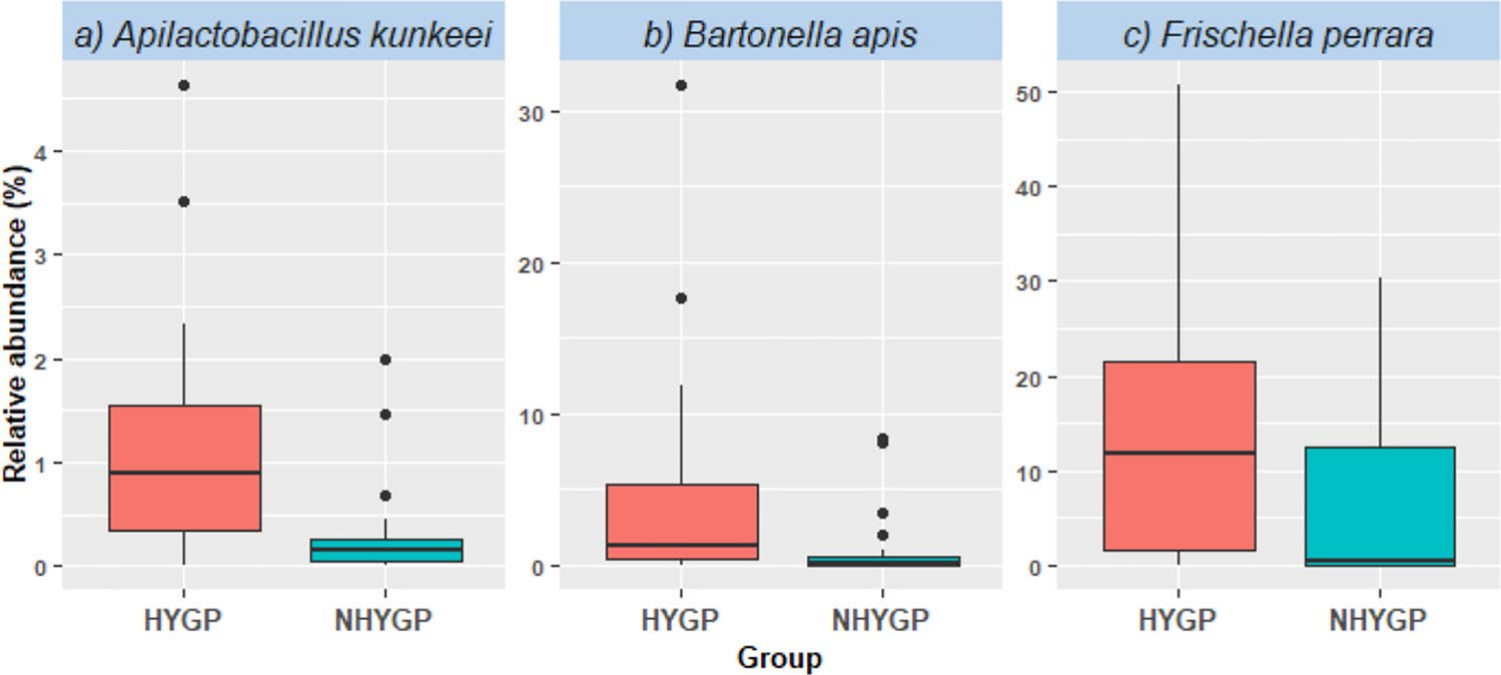
Relative abundance of three bacterial species was significantly higher in hygiene performers (HYGP) than non-hygiene performers (NHYGP). **a** *Apilactobacillus kunkeei* (*p* = 0.0439), **b** *Bartonella apis* (*p* = 0.0338), and **c** *Frischella perrara* (*p* = 0.0438)

**Fig. 3:**
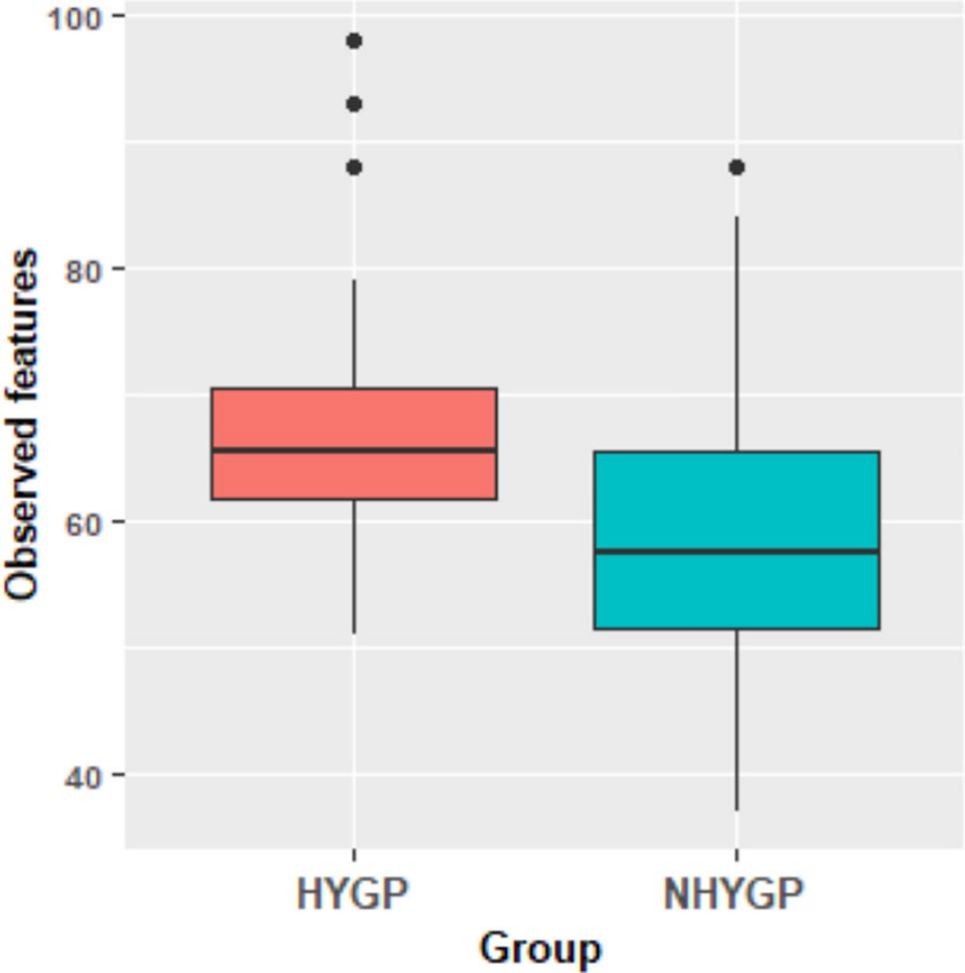
Alpha diversity, assessed by the number of amplicon sequence variants (ASVs; observed features/community richness), was significantly higher in hygiene-performing bees (HYGP) compared to non-hygiene-performing bees (NHYGP) (*p* = 0.0173)

In our comparative analysis of the gut microbiota composition between HYGP and NHYGP bees, *Apibacter mensalis,* representing 1.2% of total sequence reads, was detected exclusively in the HYGP cohort (Fig. 1) with a 40% prevalence. Among the 16 bacterial taxa assessed, three species exhibited significantly higher relative abundances in the HYGP bees, with no species demonstrating a statistically significant increase in the NHYGP bees (Fig. 2). The taxa with increased relative abundance in the HYGP bees included *Apilactobacillus kunkeei* (*p* = 0.0439), *Bartonella apis* (*p* = 0.0338), and *Frischella perrara* (*p* = 0.0438). We also analyzed the relative abundance of minor taxa, grouped collectively as “Others” (representing approximately 0.5% of all reads), which did not show significant differences among the groups. Additionally, alpha diversity, quantified by the number of observed amplicon sequence variants (ASVs) or community richness, was significantly higher in HYGP bees compared to NHYGP bees (*p* = 0.0173; Fig. 3). However, other alpha diversity indices (Evenness, Faith PD, Shannon) did not show significant differences between the groups (data not shown). Given the consistency of patterns observed across both experimental colonies and the absence of statistically significant colony effects or interactions between colony and hygienic behavior, data from the two colonies within each group were pooled for the relative abundance analysis of the bacterial species identified in this study (S1, F2).

## Discussion

Consistent with previous studies on honey bee gut microbiome, our findings showed that Proteobacteria, Firmicutes, and Actinobacteria were the dominant phyla (Martinson et al. 2011; Moran et al. 2012; Kwong and Moran 2013). However, the number of amplicon sequence variants and relative abundance of three bacterial species (*Apilactobacillus kunkeei, Bartonella apis,* and *Frischella perrara*) were significantly higher in hygiene performers (HYGP) than non-hygiene-performing (NHYGP) bees. Moreover, *Apibacter mensalis* was only present in HYGP. These findings indicate distinct differences in gut microbiome composition between HYGP and NHYGP bees, underscoring an association between microbial communities and hygienic behavior. While this study does not establish causation, it lays a foundation for future research into the potential interactions between hygienic behavior, gut microbiome, and honey bee health.

Bees performing hygienic behavior physically touch and manipulate infected brood, which exposes them to pathogens and parasites. This exposure leads to the activation of host immune response genes as a defense mechanism (Evans and Armstrong 2006). Bees that are more hygienic tend to have higher expression levels of antimicrobial peptides, such as *apidaecin* (Teixeira et al. 2021). The activation of a strong immune response is energetically costly (Paredes et al. 2011). Interestingly, the costly expression of the antimicrobial peptide *apidaecin* was reduced in bees inoculated with *Apilactobacillus kunkeei* (formerly *Lactobacillus kunkeei*) compared to microbe-deprived bees and increased bee survival by inhibiting *Serratia marcescens* (Chege et al. 2023). We speculate that *Apilactobacillus kunkeei* might be competing with pathogens directly or producing antimicrobial compounds that inhibit their proliferation in the gut, thereby reducing the need for a strong immune response from the hygienic bees.

Similarly, the most abundant bacteria species in hygiene-performing bees prime and modulate the honey bee immune system against pathogenic infections (Emery et al. 2017; Daisley et al. 2020b; Lang et al. 2022). *Bartonella apis* increased bee survival against deformed wing virus (Dosch et al. 2021). A laboratory-based experiment on hygienic behavior demonstrated the acquisition of high levels of DWV by hygiene performers during cannibalism of infected pupae (Posada-Florez et al. 2021). It is possible that hygiene performers in our study also had higher virus loads than non-performers, although this was not measured. Another abundant bacterium, *F. perrara*, is known to stimulate host immunity (Engel et al. 2015; Emery et al. 2017; Schmidt et al. 2023). *F. perrara* has been shown to increase in bees infected by *Vairimorpha* (formerly *Nosema*) *ceranae* (Sbaghdi et al. 2024) and is positively correlated with *V. ceranae* in bees fed aged pollen (Maes et al. 2016). Taken together, these beneficial gut bacteria could induce a bee's immune system, making it more efficient at responding to pathogens encountered during hygienic tasks. This may lead to a quicker and more targeted immune response, reducing the need for sustained high levels of gene expression. Further studies are needed to understand potential relationships between hygiene, pathogens, and gut microbes.

Interestingly, *Apibacter mensalis* was found only in hygiene performers and was absent in non-hygiene performers. This bacterium has been predominantly characterized in bumble bees and Asian honey bee species (*Apis cerana* and *Apis dorsata*), but is rarely found in *Apis mellifera* (Praet et al. 2016; Kwong and Moran 2016; Kwong et al. 2018; Tola et al. 2020). A recent study demonstrated that an *Apibacter* isolate can colonize the gut of *A. mellifera*, indicating the potential of host expansion (Zhang et al. 2022a). It has been suggested that *Apibacter* possesses both conserved and acquired genes important for host interaction, including those involved in amino acid synthesis and detoxification (Kwong et al. 2018; Zhang et al. 2022a). Additionally, *Apibacter* exhibited a strong negative correlation with *Crithidia bombi* and *Nosema bombi* in bumble bees (Mockler et al. 2018) and solitary bees (Fernandez De Landa et al. 2023), respectively, suggesting that *Apibacter* may play a role in enhancing bee immunity or competing with harmful pathogens. However, its correlation with other pathogens has not been documented in honey bees. Furthermore, *Apibacter* abundance has been positively correlated with foraging behavior in *Apis cerana*, although hygienic behavior was not assessed in that study (Gruneck et al. 2022). Investigating the potential association of *Apibacter mensalis* with host hygiene and protection against pathogens could provide further insights into its role in honey bee physiology and health.

Hygiene-performing bees may harbor a more diverse gut microbiome due to their involvement in larval and pupal cannibalism and frequent contact with various hive components, including brood and hive debris, although direct evidence supporting this mechanism is currently limited (Powell et al. 2014). This enhanced microbial exposure could promote increased microbial colonization and stability, potentially supporting the development of the bee’s immune system. Especially, *Apilactobacillus kunkeei* (Firmicutes) has been isolated from honey bee larvae (Vojvodic et al. 2013; Kowallik and Mikheyev 2021), pupae (Hroncova et al. 2019), and hive components (Anderson et al. 2013). Our findings indicate that this bacterial species is more abundant in hygiene performers. However, further research is needed to elucidate whether hygienic cannibalism specifically enhances the abundance of these gut microbiota in bees.

In this study, our samples were not age controlled and there is a possibility that hygiene performers and non-performers differed in age. However, a previous study on nurses and foragers showed that differences in gut microbial community composition depended upon differences in behavioral state, not age (Vernier et al. 2024), suggesting that age might not be a significant confounding factor. Furthermore, we could not infer causality in our study; a particular microbial community structure in bees could lead to heightened hygienic behavior, or conversely the act of engaging in hygienic behavior could alter the gut microbiome. Future studies should assess how specific gut microbiomes influence honey bee hygienic behavior and immune responses or vice versa, and examine whether altering microbiome composition can enhance hygiene performance in non-performers to guide interventions to improve bee health and colony resilience.

Despite the limited taxonomic resolution of our 16S rRNA sequencing approach, we were able to identify bacterial genera and species and found several potentially important differences. However, this level of resolution often lacks the power to differentiate closely related species or strains. For finer taxonomic resolution and functional characterization at the gene level, full metagenome sequencing is required. Our study shows that, despite its limitations, 16S rRNA sequencing remains effective for profiling bacterial community composition and relative abundance. Our bacterial taxonomy findings are consistent with the previous studies employing 16S rRNA sequencing (Vernier et al. 2020, Zumkhawala-Cook et al. 2024), indicating that the ecological patterns and trends identified are robust and contribute valuable insights to the field.

In conclusion, our study demonstrates that hygiene performers have a higher relative abundance of some members of their core bacterial communities compared to non-hygiene performers. Hygiene performers also had a phylum of gut microbiota that was not present in non-hygiene performers. These findings provide interesting and novel insights into hygienic behavior and the gut microbes of hygienic bees and suggest the need for additional studies to understand whether and how these bacteria contribute to overall colony hygiene and health. The results of this study may inform ongoing work to develop beneficial probiotic supplements for honey bees and other social insects (Motta et al. 2022). Additionally, the study identifies candidate taxa for future functional investigations in bee hygienic behavior and host protection against pathogens. Further work is needed to fully understand the role of these species in honey bee health.

## Supplementary Information

Below is the link to the electronic supplementary material.

Supplementary file1 (XLSX 29 KB)

## Data Availability

The raw data that support the findings of this study are available upon reasonable request.
